# Description of the 180° Rotational Mucosal Flap Technique in Patients With Incomplete Cleft Palate: A Study From Cali, Colombia

**DOI:** 10.7759/cureus.114671

**Published:** 2026-08-17

**Authors:** Samuel G Calderón Crespo, Angélica M Franco Cárdenas, Juan J Zorrilla Ariza, Valentina Ulloa Zuluaga, Diego J Caycedo García

**Affiliations:** 1 Plastic and Reconstructive Surgery, Universidad del Valle, Cali, COL; 2 School of Medicine, Universidad del Valle, Cali, COL; 3 PLASTICUV, Universidad del Valle, Cali, COL; 4 School of Medicine, Universidad Autónoma de Bucaramanga, Bucaramanga, COL; 5 School of Medicine, Instituto Colombiano de Estudios Superiores de Incolda (ICESI), Cali, COL; 6 School of Medicine, Pontificia Universidad Javeriana Cali, Cali, COL; 7 Craniofacial Surgery, Fundación Universitaria de Ciencias de la Salud, Bogotá, COL; 8 Plastic and Reconstructive Surgery, Universidad de Buenos Aires, Buenos Aires, ARG; 9 School of Medicine, Fundación Universitaria del Norte, Barranquilla, COL

**Keywords:** cleft lip and palate surgery, cleft palate repair, congenital palatal fistula, palatal flap, palatoplasty, palatorrhaphy

## Abstract

Objectives

This study aims to provide a detailed, reproducible description of a 180° rotational mucosal flap technique from the oral to the nasal layer for palatal reconstruction in patients with incomplete cleft palate and to report the early clinical outcomes associated with its use, including total operative time and the occurrence of secondary palatal fistulas during follow-up.

Materials and methods

A retrospective descriptive case series was conducted at a referral institution in Cali, Colombia, between January 2021 and August 2025. A total of 84 consecutive patients with incomplete cleft palate (Veau classifications I and II) who underwent primary palatorrhaphy using a 180° rotational mucosal flap were evaluated. The surgical approach incorporates a triangular oral mucosal flap with a 45° apex elevated, rotated 180°, and sutured into the nasal mucosal plane to provide a watertight reinforcement at the hard-soft palate junction. Clinical outcomes, operative time, and complications were analyzed through medical records over a 36-week follow-up period.

Results

The study cohort comprised 63 males (75%) and 21 females (25%). The mean total surgical duration was 60.0 minutes (standard deviation: 5.3 minutes). Ernst relaxation incisions were required in 55 patients (65.4%) to achieve a tension-free closure. During the 36-week follow-up period, complete cleft closure at the hard-soft palate junction was achieved in all cases, with no documented secondary palatal fistulas.

Conclusions

The 180° rotational mucosal flap technique was reproducible, achieving complete anatomical cleft closure in all patients, with no secondary palatal fistulas observed during the 36-week follow-up period.

## Introduction

Globally, cleft lip and palate anomalies are observed in 4.76 to 23.79 per 10,000 births. Cleft palate, also known as palatal cleft, has an incidence ranging from 1.3 to 25.3 per 10,000 births [1]. Cleft palate, embryologically classified as a secondary palate defect, is a congenital anomaly that can significantly impact a child's oral communication, feeding, and hearing capabilities [2]. Palatal reconstruction, performed through a specialized surgical procedure, aims to correct this specific condition [3]. Notably, the junction between the hard and soft palate is the zone most prone to developing fistulas or dehiscence following surgical intervention [4].

Within the framework of this scientific research, a reproducible surgical technique for palatal reconstruction is proposed. This technique is aimed at increasing the probability of achieving optimal outcomes and offering greater reliability in preventing the formation of residual palatal fistulas through its distinctive anatomical and surgical features. The objective of this study is to describe a reproducible description of a 180° rotational mucosal flap technique from the oral to the nasal layer for palatal reconstruction in patients with incomplete cleft palate and to report the early clinical outcomes associated with its use, including operative time and the occurrence of palatal fistula during follow-up.

## Materials and methods

A retrospective descriptive case series and literature review were conducted at a referral institution, Hospital Universitario del Valle "Evaristo García" E.S.E. in Cali, Colombia, between January 2021 and August 2025. A total of 84 consecutive patients diagnosed with incomplete cleft palate were included. All patients underwent primary palatorrhaphy using an alternative technique based on multilayer mucosal flap reconstruction (180° rotational mucosal flap), developed by the senior author, and were followed for a total of 36 weeks.

This surgical approach incorporates a triangular oral mucosal flap with a 45° apex rotated toward the nasal layer. The flap was designed based on a comprehensive review of the literature and aimed to provide a watertight reinforcement at the junction of the hard and soft palate, thereby reducing the risk of residual palatal fistula formation.

Study population

Inclusion criteria consisted of patients diagnosed with an incomplete cleft palate (Veau classifications I and II) who underwent surgical correction via primary palatorrhaphy [5]. Exclusion criteria comprised patients whose post-surgical treatment was incomplete or those who were lost to clinical follow-up. Anatomical and functional variables, as well as clinical outcomes, were defined and evaluated through a retrospective analysis of medical records. All legal guardians of the patients provided written informed consent for participation in the study and the use of photographic records, ensuring that data collection strictly complied with the ethical principles of the Declaration of Helsinki.

Statistical analysis

The collected data were strictly anonymized to ensure patient confidentiality and subsequently tabulated in a database using Microsoft Excel software, version 16.16.11 (Microsoft Corp., Redmond, WA). Descriptive statistical tools were utilized to process and summarize the study variables. Qualitative and dichotomous variables (such as sex, cleft classification, the need for Ernst relaxation incisions, and the presence or absence of secondary fistulas) were expressed using absolute frequencies (n) and percentages (%). Concurrently, continuous quantitative variables (such as patient age, total operative time, and the specific flap fabrication time) were reported using measures of central tendency and dispersion, specifically means, ranges, and averages, as appropriate. Due to the intrinsic nature of the methodological design of this case series, the selected sample size (n = 84), and the absence of a comparison or control group, inferential statistical tests and probability significance calculations were not applied.

Surgical technique 

**Figure 1:**
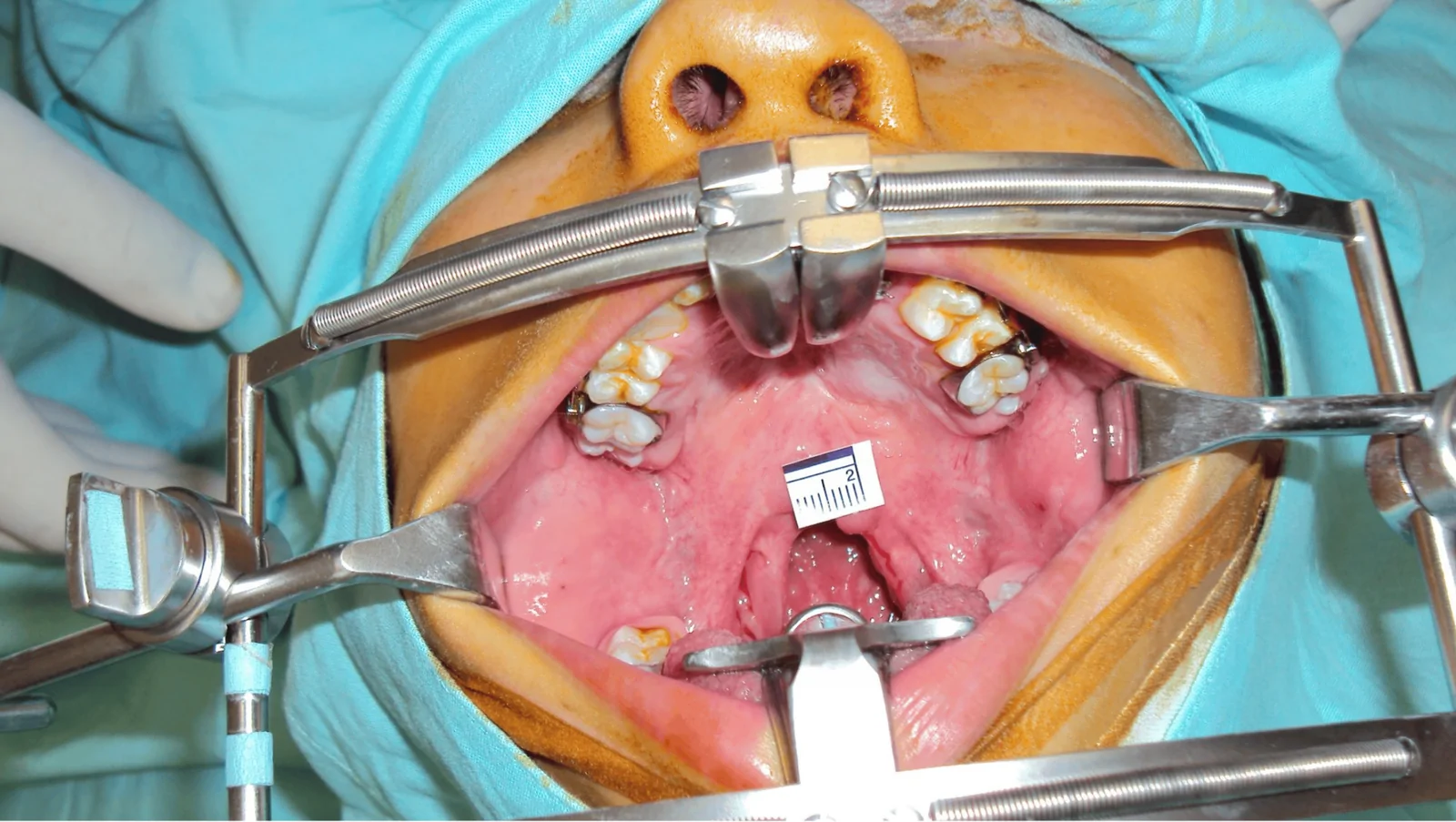
Intraoperative placement of the Dingman mouth gag to facilitate access and visualization (case 1).

The surgery was performed under general anesthesia. A Dingman mouth gag (Ref: 38-100-00-jimar, Surgiwell, Tuttlingen, Alemania) was used to facilitate intraoral access and visualization (Figure 1). Infiltration with bupivacaine and epinephrine was performed to control pain and hemorrhage. The suture used was 5-0 polyglactin with a half-circle taper needle (RB-1) (Ref: Sut-Propyl-Acid-5/0, Natural Master, United Medical Supplies, China).

**Figure 2:**
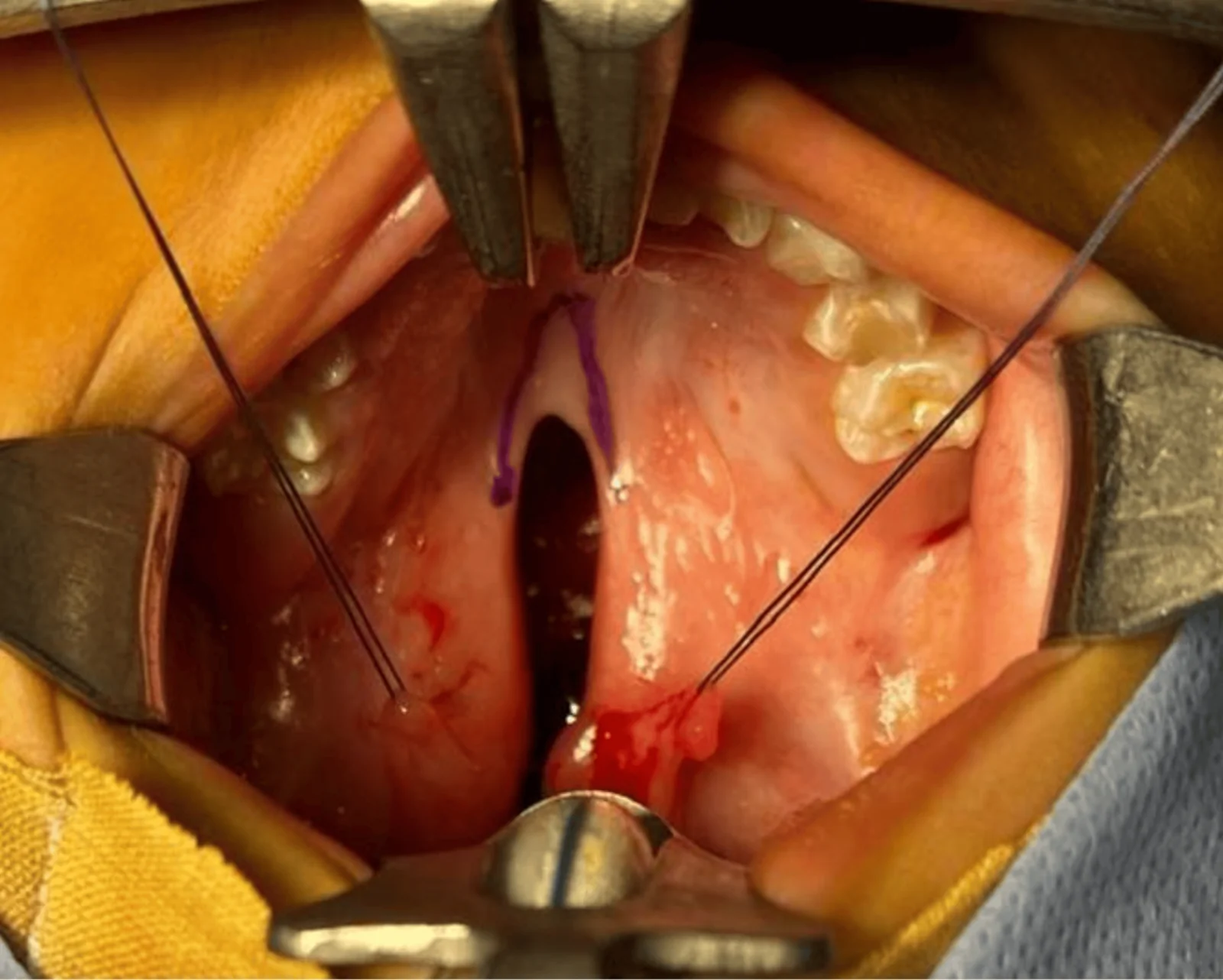
Design of a triangular oral mucosal flap with a 45° apex, incised using a number 15 scalpel blade (case 1).

**Figure 3:**
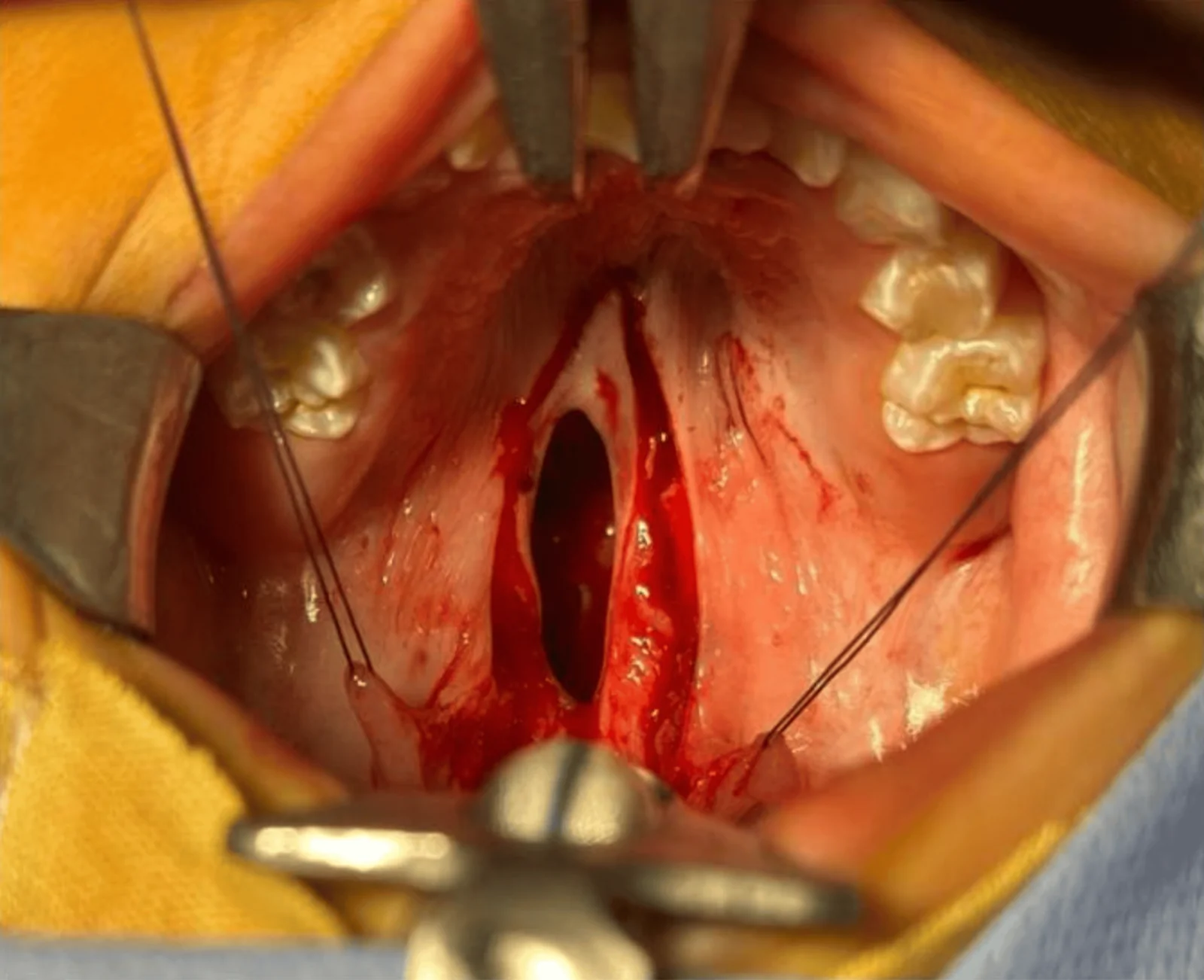
Elevation and controlled dissection by layers (case 1).

**Figure 4:**
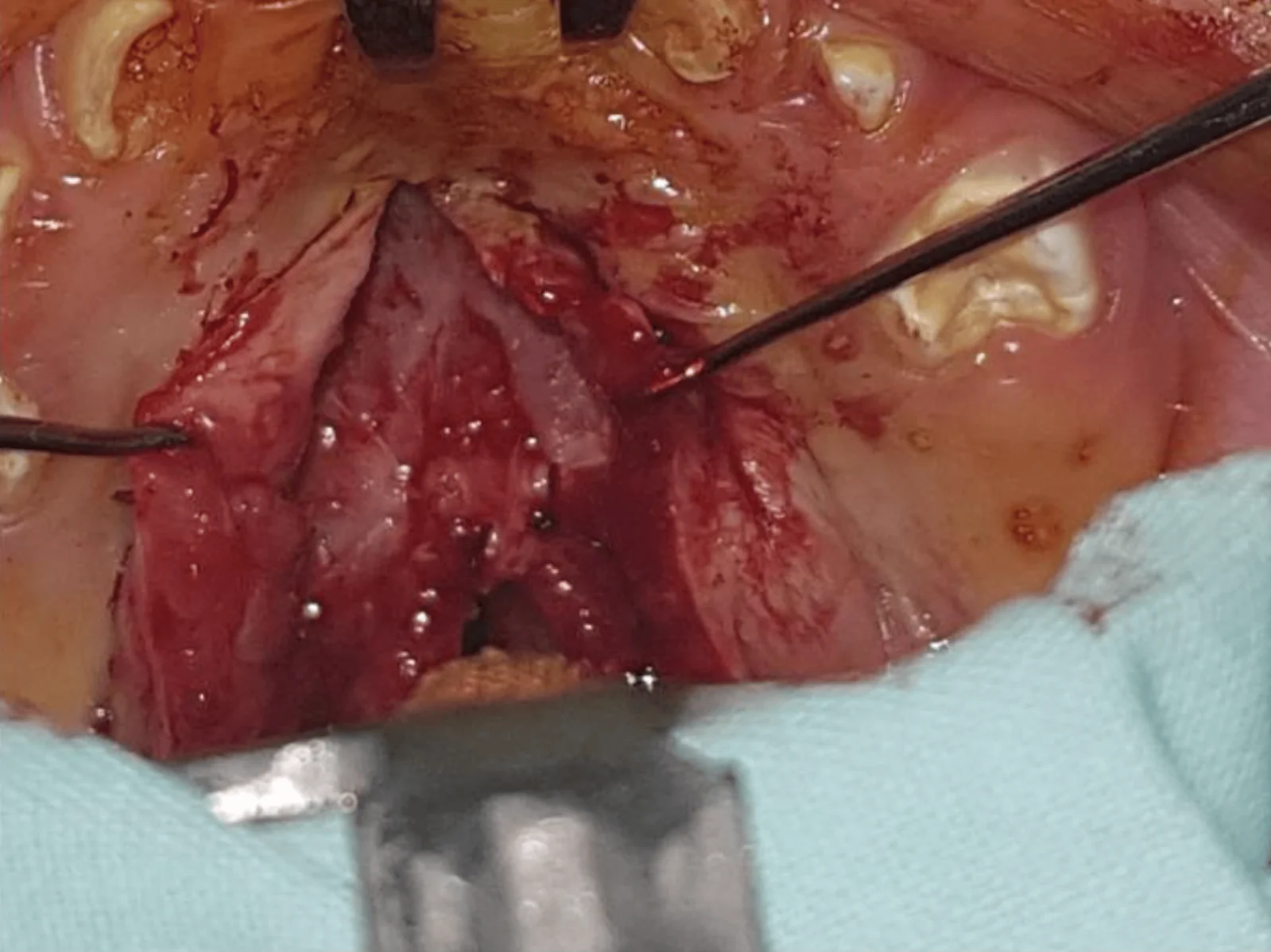
180° rotation from the non-raw oral side toward the nasal mucosal plane, sutured with 5-0 polyglactin using a round taper needle (case 1).

**Figure 5:**
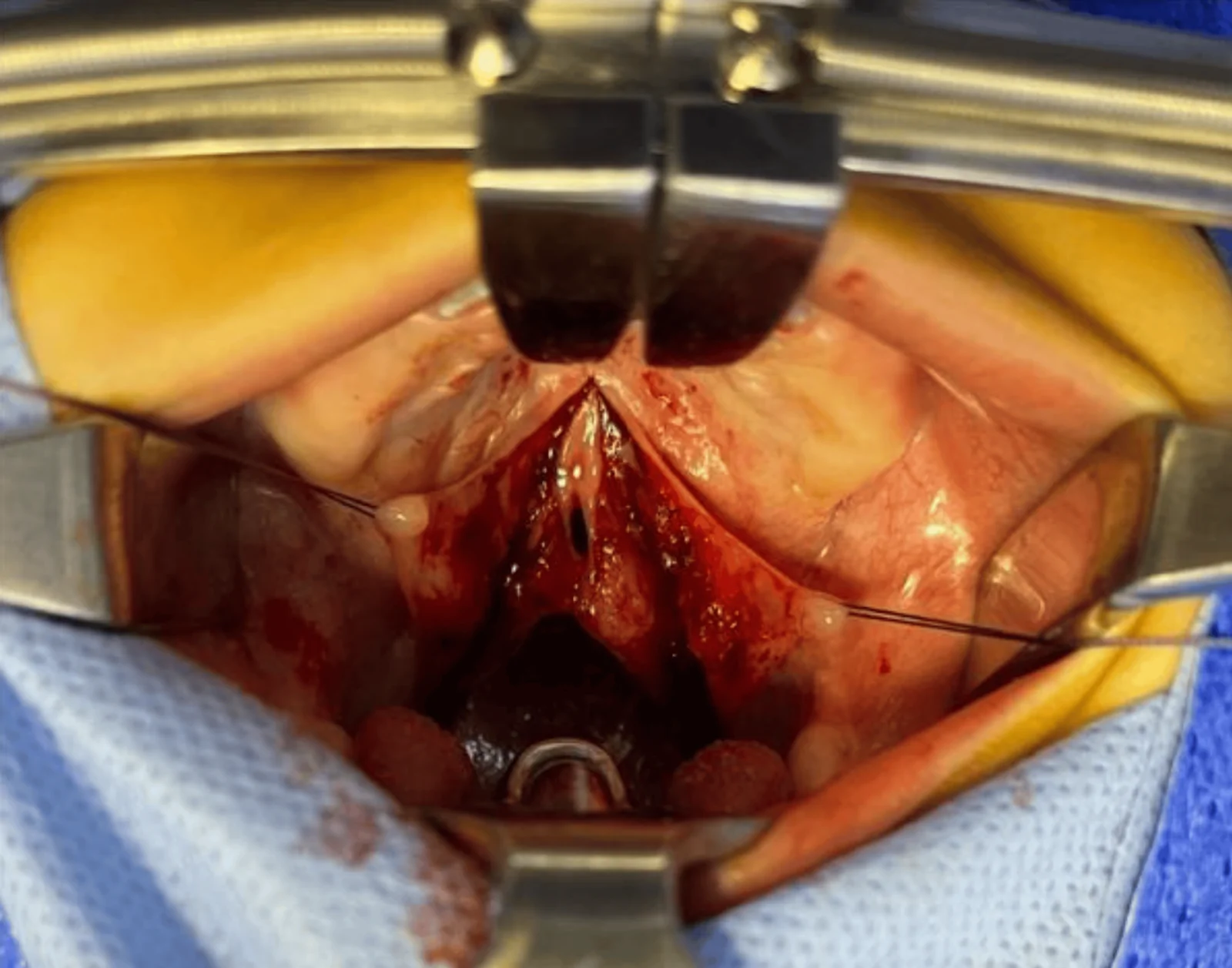
Design of a triangular oral mucosal flap with a 45° apex (case 2).

**Figure 6:**
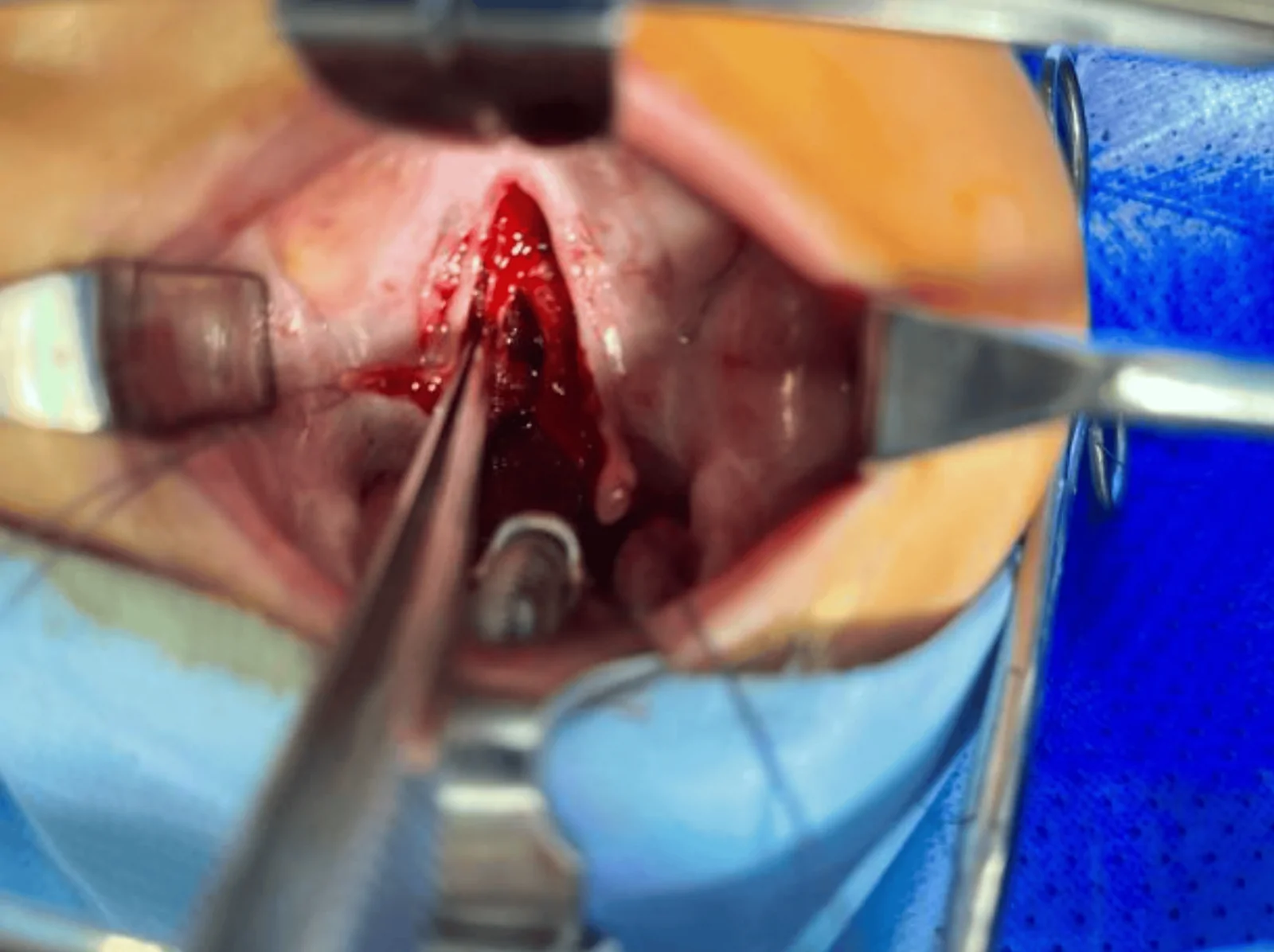
Controlled layer-by-layer elevation and dissection (case 2).

**Figure 7:**
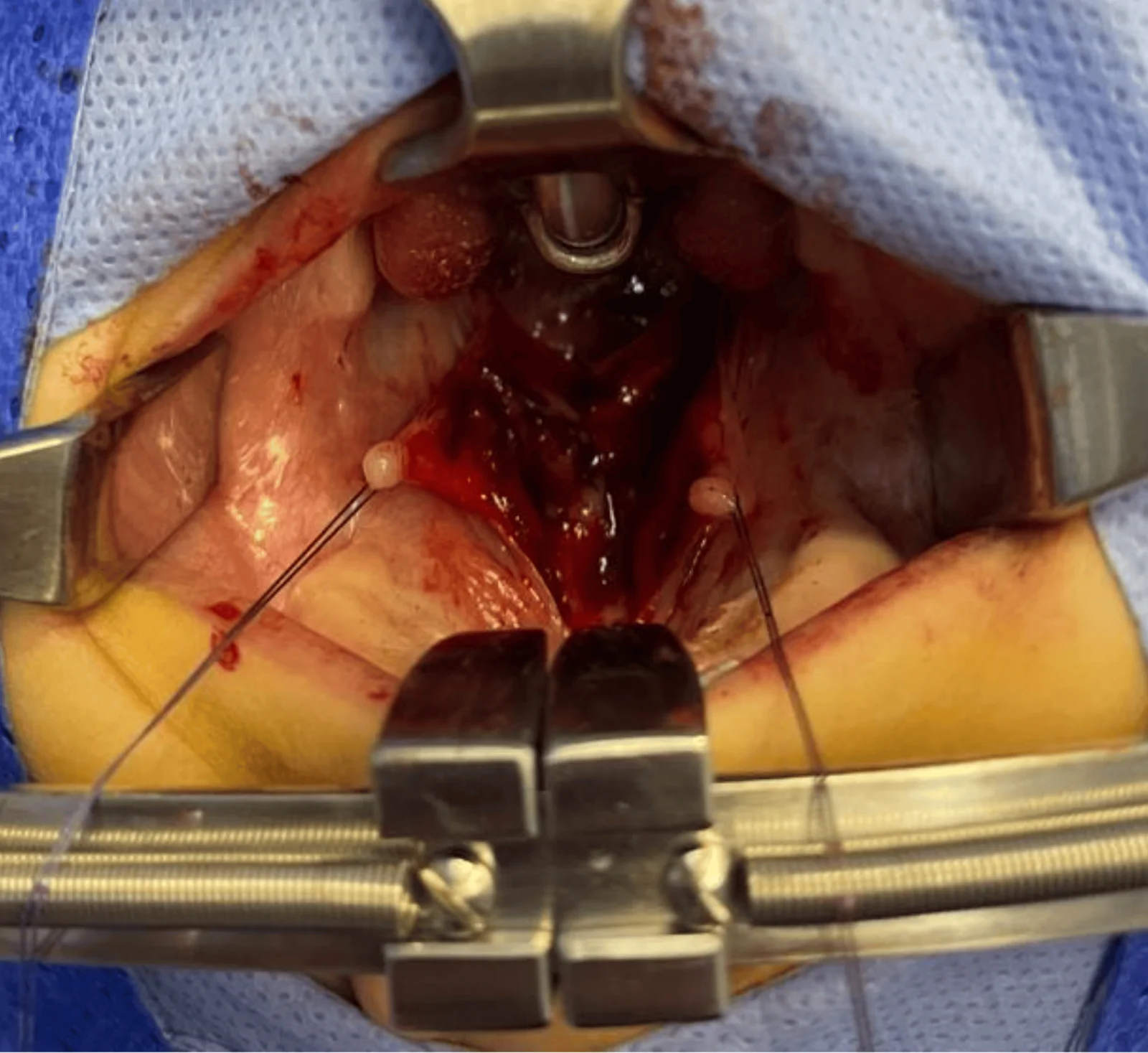
180° rotation (case 2).

**Figure 8:**
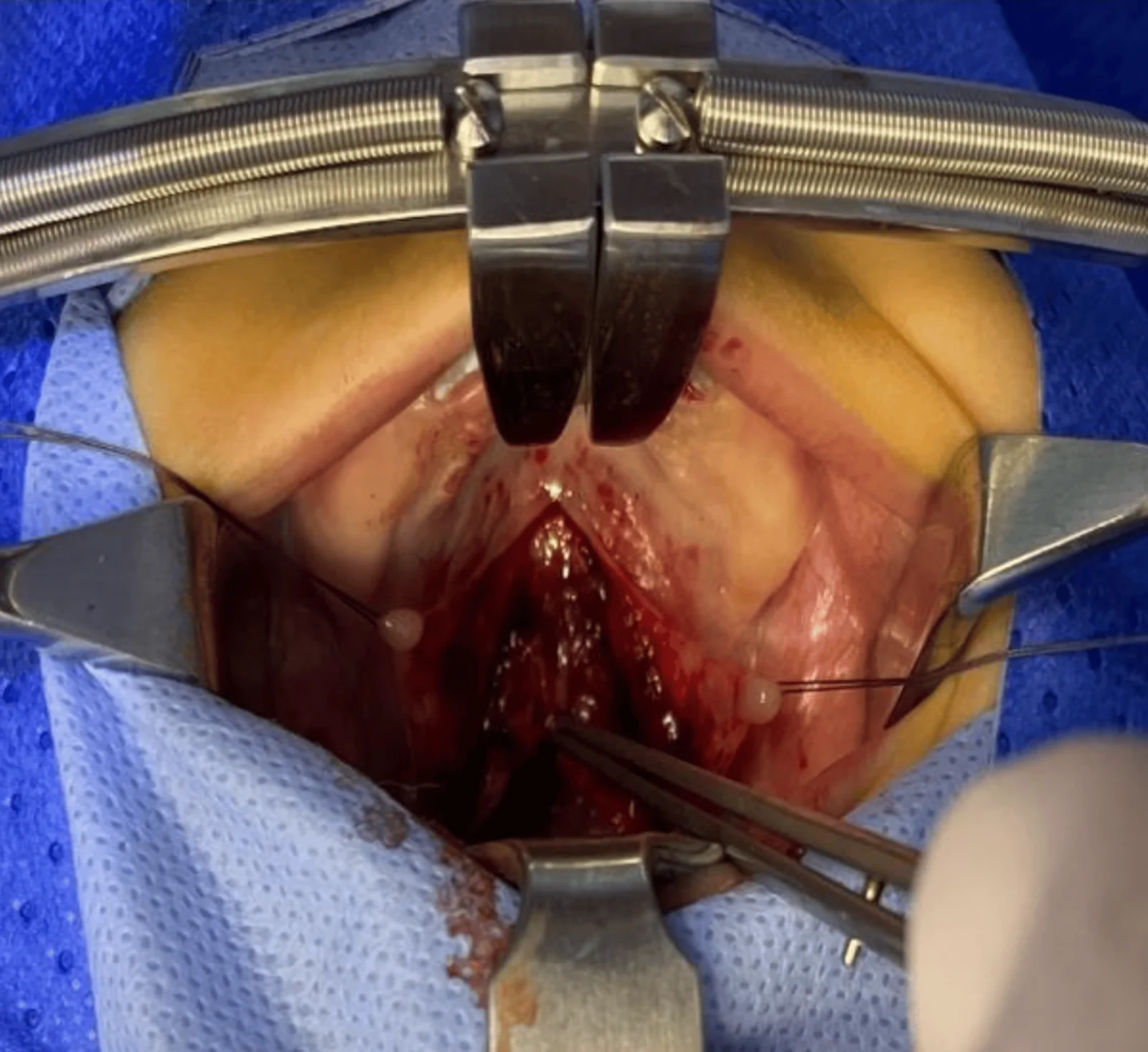
Polyglactin 5-0 sutures with a round needle (case 2).

**Figure 9:**
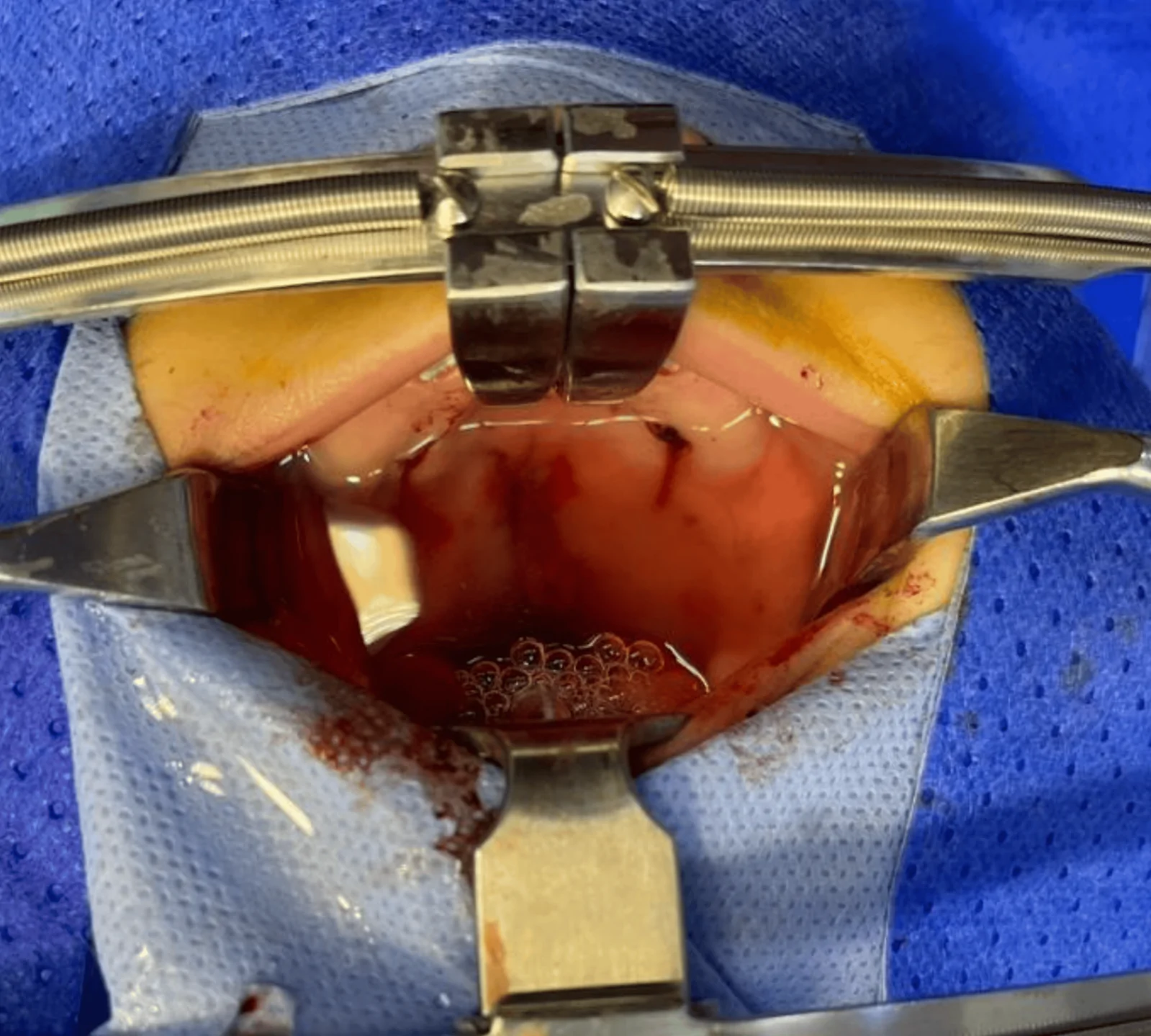
Hermetic seal verified via fluid accumulation test (case 2).

To prevent fistula formation at the junction of the soft palate, an alternative surgical technique based on layered mucosal flap reconstruction was utilized. Once the surgical approach was completed, a triangular oral mucosal flap with a 45° apex was designed, elevated, and, under controlled dissection, freed and rotated 180° from the non-raw oral side toward the nasal mucosal plane. It was then sutured into this same plane using 5-0 polyglactin with a round taper needle. The closure was performed in layers, achieving complete closure of the defect; figures from two cases will be presented (Figures 2-9).

**Figure 10:**
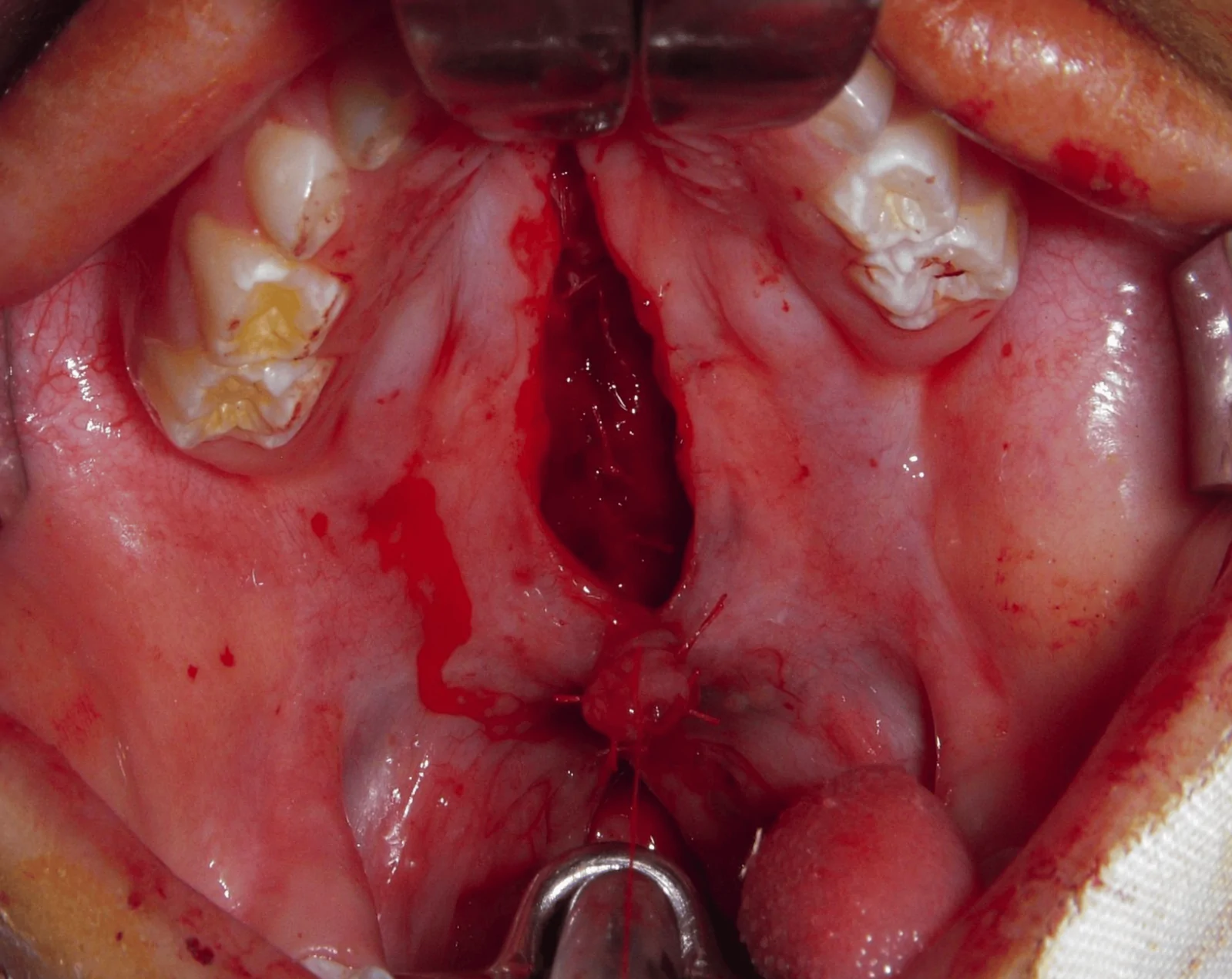
Sequential steps to complete the palatorrhaphy using the Sanvenero-Rosselli intravelar veloplasty technique (case 3).

**Figure 11:**
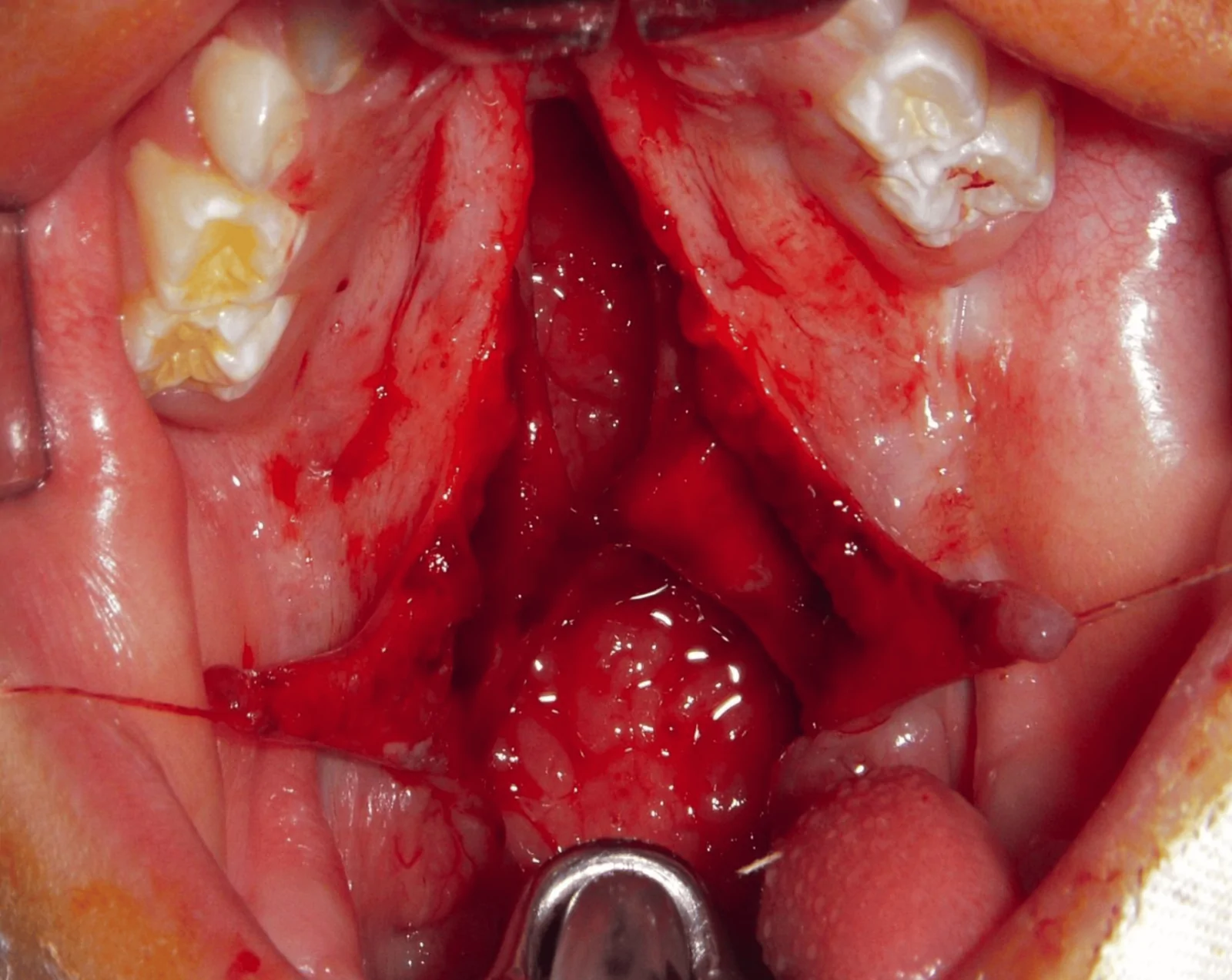
Sequential steps to complete the palatorrhaphy using the Sanvenero-Rosselli intravelar veloplasty technique (case 3).

**Figure 12:**
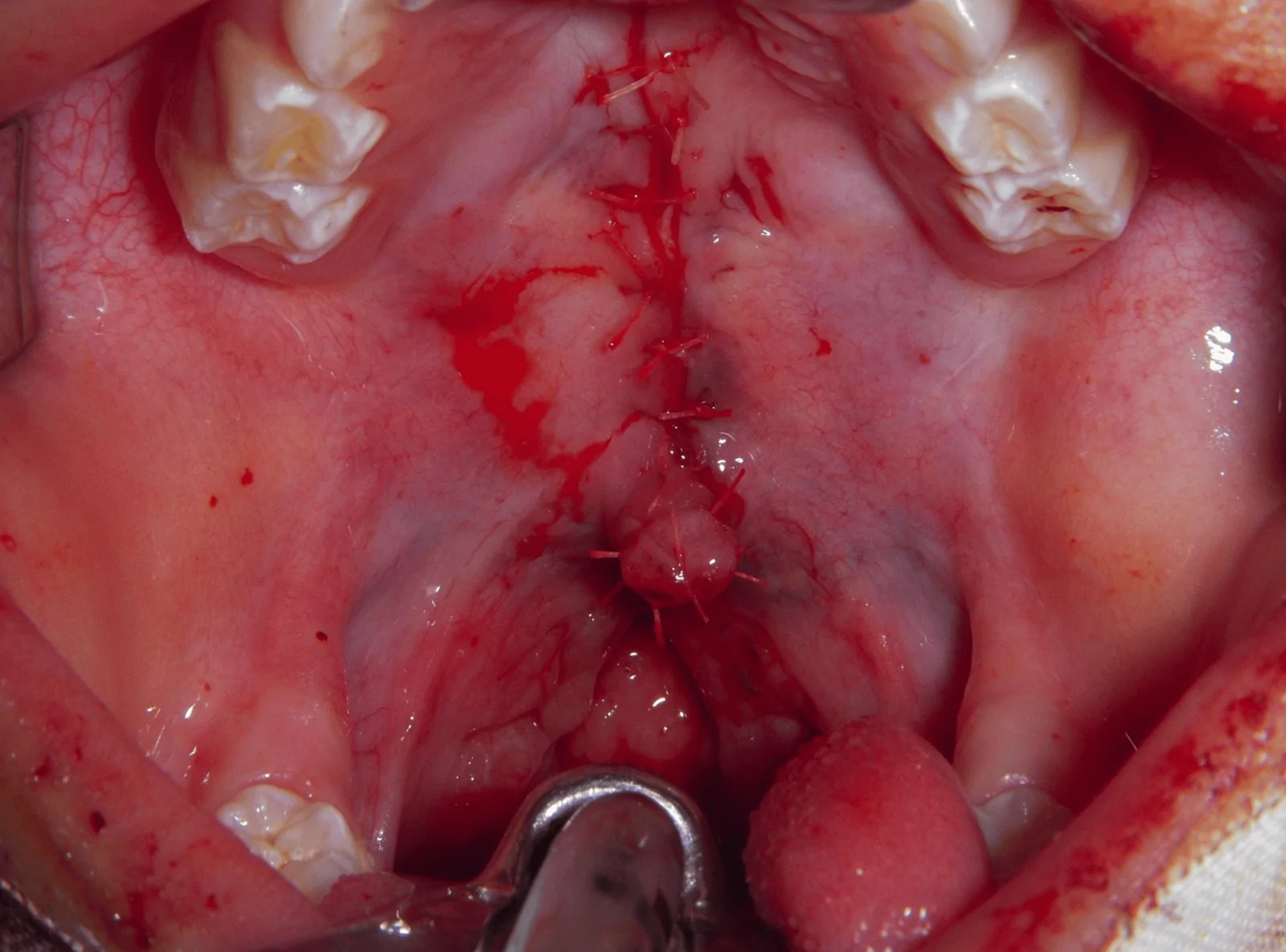
Anteroposterior elongation of the soft palate by 10 mm achieved using the palatopharyngeus muscles (case 3).

**Figure 13:**
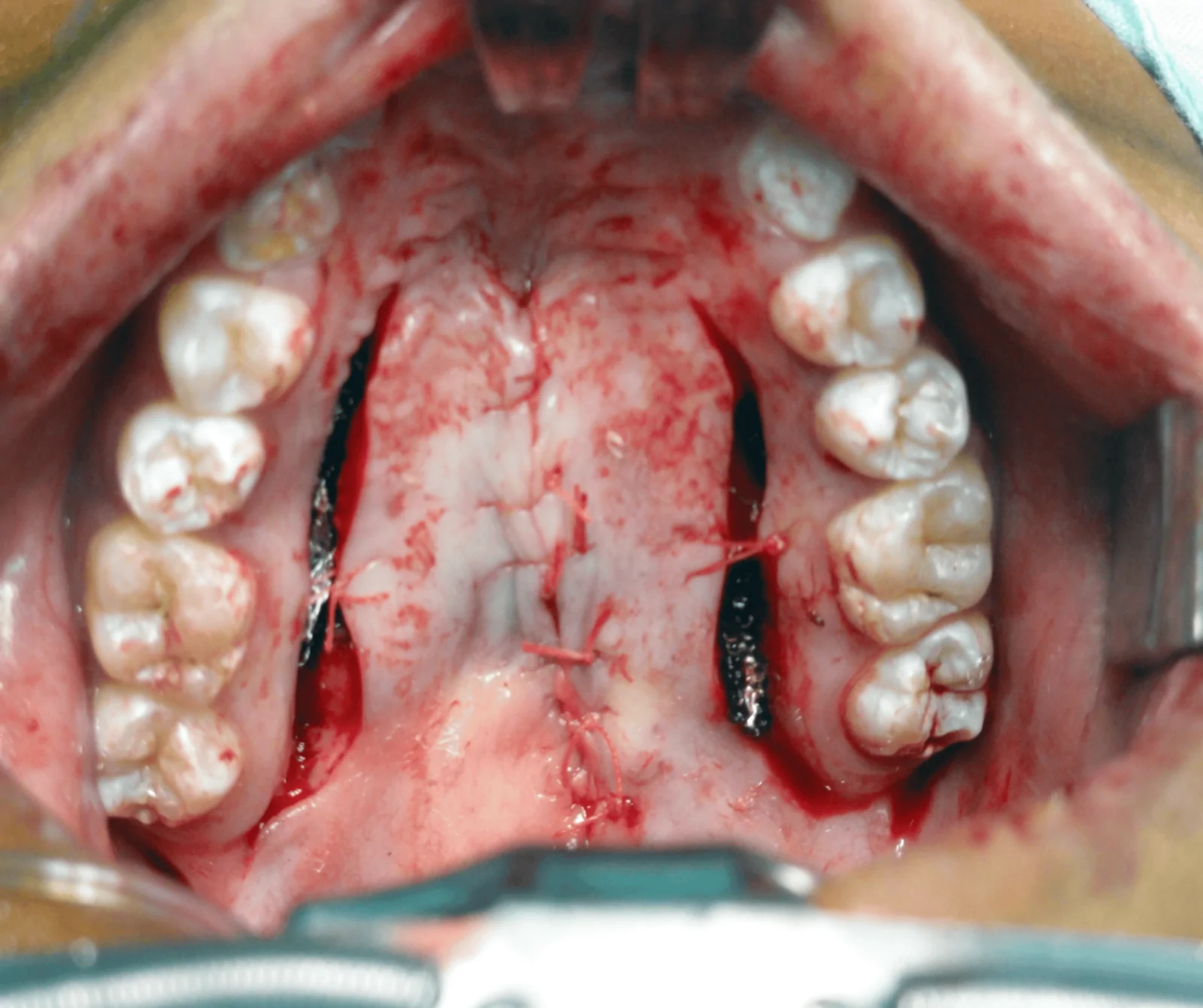
Relaxation incisions shown in the Ernst retromolar space, allowing for tension-free closure of the midline (case 3).

The primary palatorrhaphy procedure was completed using the Sanvenero-Rosselli intravelar veloplasty technique in three layers: nasal mucosa, muscular layer, and oral mucosa. In cases where the cleft width was greater than 15 mm, it was necessary to perform Ernst relaxation incisions in the retromolar space to achieve tension-free closure, or, in some cases, to perform the von Langenbeck palatoplasty technique (Figures 10-13).

Additional considerations 

Given that the surgery is intraoral, the positioning of the orotracheal tube was carefully managed during the procedure. The orotracheal tube was positioned orally, keeping in mind that the subsequent placement of the Dingman mouth gag could compress the tube against the tongue and the muscles of the floor of the mouth. An armored (reinforced) endotracheal tube was used to facilitate the placement of the retractor without risk of kinking or collapsing.

The Dingman mouth gag provided the advantage of superior cheek retraction, which helped improve intraoral exposure and maintain an accessible surgical field. Furthermore, it protected the orotracheal tube and served as a suture holder.

Postoperative care 

Patients were discharged on the same day of surgery and attended their first postoperative follow-up visit one week after the procedure. Subsequent evaluations were performed at four, 12, and 36 weeks postoperatively. Caregivers were instructed to maintain a liquid diet without the use of bottles or pacifiers for the first two weeks, followed by a semisolid diet during the third postoperative week.

## Results

Between January 2021 and August 2025, reconstruction was performed on 63 male and 21 female patients using a mucosal flap at the junction of the hard and soft palate, with a 180-degree rotation of the oral mucosal plane toward the nasal plane (Table 1).

**Table 1:** Patient data and outcomes.

Data	Characteristics	Number of Patients (n = 84)	Percentage (%)
Sex	Male	63	75.0%
	Female	21	25.0%
Age	12–18 months	52	61.9%
	19 months to 5 years	18	21.4%
	> 5 years	14	16.7%
Palatorrhaphy Technique	Sanvenero-Rosselli	29	34.5%
	Sanvenero-Rosselli + von Langenbeck	55	65.5%

The surgical procedure had a mean duration of 60.0 minutes (standard deviation: 5.3 minutes) for the entire operation, including the 180° rotational flap design. Ernst relaxation incisions in the retromolar space were required in 55 patients (65.4%) to achieve a tension-free closure.

During the 36-week follow-up period, a 100% cleft closure at the hard-soft palate junction was achieved in all cases, without documentation of secondary palatal fistulas.

**Figure 14:**
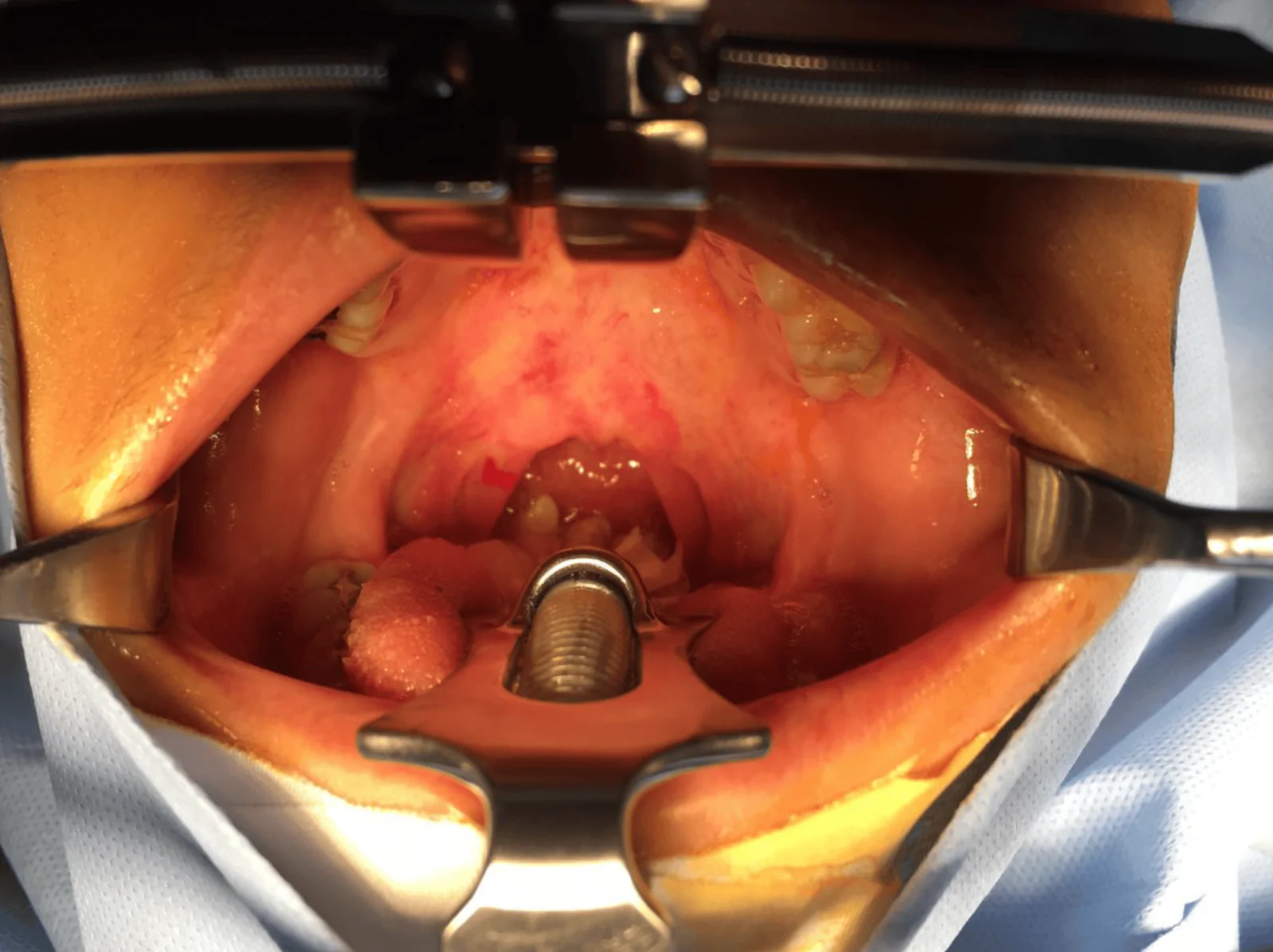
Patient who required uvuloplasty (case 4). Result observed 12 months post-palatorrhaphy. In this case, a patient with an unsatisfactory uvular morphology underwent surgical intervention. If the uvula is not properly repaired and remains bifid or amputated, parents express concern and perceive it as a surgical failure. Therefore, a revision procedure was performed to repair the uvula in two layers. Two or three mattress sutures should be utilized to achieve optimal border approximation.

All patients followed an interdisciplinary follow-up protocol at the cleft lip and palate clinic, which included speech therapy, otolaryngology, and pediatric dentistry, among others. To date, 30 patients have undergone alveolar bone grafting, and seven patients required uvuloplasty to modify the shape or size of the uvula (Figure 14).

## Discussion

The present case series documented complete cleft closure in all 84 patients with incomplete cleft palate (Veau I-II) treated with the 180° rotational mucosal flap technique, with no documented cases of secondary palatal fistula over a 36-week follow-up period. Historically, the risk of fistula development following palatorrhaphy has been reported to range as widely as 3% to 45% [6-8]. However, more recent and methodologically robust data suggest a considerably narrower and lower range: a large prospective multicenter observational study of 1,178 infants reported an overall fistula incidence of 5.3% [9]; a retrospective cohort of 505 patients reported an incidence of 10.1% [10]; and a systematic review pooling 4,651 patients across 36 studies reported a mean fistula rate of 9.94% [11]. Given the methodological strength of these more recent, larger, and in some cases prospective studies, the 0% fistula rate observed in the present series should be benchmarked primarily against this narrower and better-supported range rather than against the wider historical extremes.

Regardless of the technique utilized, the highest incidence of fistulas has traditionally been reported in patients with previous palatal surgeries [12], and once an oronasal fistula is repaired, the recurrence risk has been reported to vary between 37% and 50% [13]. Although various factors related to secondary fistulas have been evaluated, it is widely recognized that the surgical technique used, suture tension, bleeding, and infection can increase the risk of this type of complication. Certain studies, such as that by Emory et al., describe a lower fistula risk in patients operated on before 12 months of age [14]; while no patient in the present series underwent surgery prior to 12 months of age, no fistula formation was documented during follow-up, a finding that runs counter to this previously described age-related pattern and may instead reflect the anatomical reinforcement provided by the flap itself rather than the timing of surgery.

A 2014 study reported an overall fistula rate of 17.7% among all patients but described a lower occurrence of fistulas when a rotational palatorrhaphy technique was used compared to the V-Y (Veau-Wardill-Kilner) pushback technique [15]. It is important to note that techniques such as the V-Y pushback often leave anterior fistulas when attempting to retroposition tissue posteriorly, whereas in techniques like the Furlow double-opposing Z-plasty, non-union palatal fistulas may appear because these transposition zones are subjected to higher tension [16]. The 180° mucosal flap rotation technique described herein is designed to avoid fistula formation at both of these locations, as it provides a watertight seal and reinforcement specifically at the site of maximum tension between the hard and soft palate.

The favorable outcome reported here is further supported by several strengths of the technique itself. It is described with an unusual degree of surgical detail, covering flap design, mouth gag selection, endotracheal tube management, and suture technique, and is illustrated through sequential intraoperative photography, which enhances the reproducibility of the procedure for surgeons experienced in cleft palate surgery. Notably, this anatomical reinforcement does not appear to compromise operative efficiency. As reported in the results, the mean total procedure time was 60 minutes, suggesting that flap fabrication does not substantially prolong the overall surgical procedure while providing potential anatomical and reconstructive benefits.

It is considered that the proposed technique does not increase the risk of velopharyngeal insufficiency (VPI), although this was not objectively evaluated in the present series. A preemptive measure against VPI occurrence is the use of intravelar veloplasty with pharyngoplasty via the Sanvenero-Rosselli technique, which successfully elongates the soft palate and helps prevent difficulties in separating the nasopharynx from the oropharynx during swallowing, nasal regurgitation, and hypernasal speech [17]. This concern is further contextualized by a systematic review and meta-analysis of 32 studies, which found that VPI risk also varies meaningfully by surgical technique, with the Furlow double-opposing Z-plasty associated with a significantly lower risk of VPI compared with the Bardach repair (RR 0.41, 95% CI 0.23-0.71) [18]. This reinforces that VPI risk, like fistula risk, is technique-dependent and warrants dedicated, validated assessment in future evaluations of the 180° rotational flap.

Key factors for an adequate surgical outcome include the surgeon's experience and adherence to core technical principles: adequate mobilization of all flaps (both mucosal and mucoperiosteal) to ensure a tension-free closure, careful tissue handling, particularly at the medial borders of the cleft, and the use of high-tensile, fine-gauge sutures with round taper needles to minimize tissue trauma. The importance of surgeon-specific factors is corroborated by more recent evidence: a large prospective multicenter study found that observed fistula rates ranged from 0% to 30% across individual surgeons, a spread considerably wider than the 5.7% difference attributable to surgical technique, and that this variation was independent of years in practice or annual surgical volume [9]. Similarly, an independent retrospective cohort found individual surgeons to be a significant predictor of fistula occurrence, with an odds ratio as high as 6.32 between the highest- and lowest-performing surgeons, alongside a significant protective effect of higher cumulative surgeon volume [10]. Because all procedures in the present series were performed by a single experienced surgeon, this enhances internal technical consistency but limits external validity and raises the possibility that the favorable outcomes reflect a surgeon-specific or learning-curve effect rather than a purely technique-specific one.

Limitations

The current study describes a case series of modest size relative to large multicenter cohorts, with short-to-medium-term outcomes regarding fistula occurrence following the exclusive use of the 180° mucosal flap in primary palatorraphy for incomplete cleft palate. Several additional limitations should be acknowledged more explicitly. First, the study is retrospective, single-center, and lacks a comparison group; consequently, the absence of fistula formation cannot be attributed solely to the flap technique, and no claim of superiority over established techniques can be supported by these data alone. Second, the series included only patients with incomplete cleft palate (Veau I-II), a subgroup with substantially lower baseline fistula risk than more severe clefts. The present findings should therefore not be generalized to more severe cleft types. Third, although speech and feeding outcomes were reported as satisfactory, they were not assessed using validated objective measures such as standardized speech evaluation, velopharyngeal competence assessment, or patient-reported outcome instruments. Fourth, the single-surgeon design, discussed above, limits external validity. Fifth, the 36-week follow-up period is shorter than the follow-up windows used in much of the comparative literature, raising the possibility that some fistulas, particularly smaller or asymptomatic ones, were not yet clinically apparent at the time of final assessment. Future studies could address these limitations by incorporating control groups or randomization between patients treated with and without the 180° mucosal flap, extending inclusion to complete (Veau III-IV) clefts, involving multiple surgeons, and incorporating validated speech and velopharyngeal outcome measures.

## Conclusions

In this series of cases, the 180°-rotated mucosal flap technique proved to be reproducible, achieving anatomical layer-by-layer closure in all patients who underwent surgery, with no occurrence of secondary palatal fistula during the 36-week follow-up. These preliminary findings suggest that the technique is feasible and safe for the primary correction of incomplete cleft palate. Prospective comparative studies are needed to confirm these results.
